# Deep Learning Based Fire Risk Detection on Construction Sites

**DOI:** 10.3390/s23229095

**Published:** 2023-11-10

**Authors:** Hojune Ann, Ki Young Koo

**Affiliations:** Vibration Engineering Section, Faculty of Environment, Science, and Economics, University of Exeter, Exeter EX4 4QF, UK; ha485@exeter.ac.uk

**Keywords:** deep learning, ignition sources, combustible materials, object detection, computer vision, Yolov5, EfficientDet, fire risk detection, construction sites, fire safety

## Abstract

The recent large-scale fire incidents on construction sites in South Korea have highlighted the need for computer vision technology to detect fire risks before an actual occurrence of fire. This study developed a proactive fire risk detection system by detecting the coexistence of an ignition source (sparks) and a combustible material (urethane foam or Styrofoam) using object detection on images from a surveillance camera. Statistical analysis was carried out on fire incidences on construction sites in South Korea to provide insight into the cause of the large-scale fire incidents. Labeling approaches were discussed to improve the performance of the object detectors for sparks and urethane foams. Detecting ignition sources and combustible materials at a distance was discussed in order to improve the performance for long-distance objects. Two candidate deep learning models, Yolov5 and EfficientDet, were compared in their performance. It was found that Yolov5 showed slightly higher mAP performances: Yolov5 models showed mAPs from 87% to 90% and EfficientDet models showed mAPs from 82% to 87%, depending on the complexity of the model. However, Yolov5 showed distinctive advantages over EfficientDet in terms of easiness and speed of learning.

## 1. Introduction

**Figure 1:**
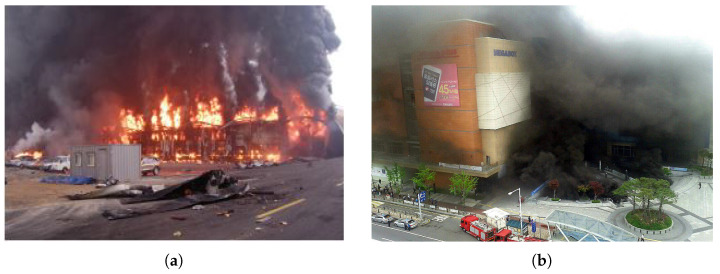
Fire accident cases on construction sites (**a**) Icheon Refrigerated Warehouse site 2008. (**b**) Goyang Bus Terminal site 2014.

Fires on construction sites, whether they are new or undergoing refurbishment, are infrequent but can have severe and devastating consequences. South Korea has witnessed several large-scale fire incidents on construction sites, as illustrated in Figure 1. For instance, at the Icheon Refrigerated Warehouse construction site, the ignition of oil vapour during a urethane foaming operation, caused by an unidentified source, led to a fire. Similarly, at the Goyang Bus Terminal construction site, the ignition of urethane foam by sparks from welding work resulted in 7 deaths and 41 injuries. These incidents exemplify the common characteristic of catastrophic fires on South Korean construction sites, where a heat source (typically welding) and highly combustible materials (such as urethane foam or Styrofoam used for insulation) are in close proximity during various stages of construction.

The condition is prevalent in South Korean construction sites, particularly during the final stages, as multiple construction activities take place simultaneously within confined building floors with the aim of reducing construction times and costs. However, this poses significant fire hazards and requires careful management to prevent such devastating incidents.

**Figure 2:**
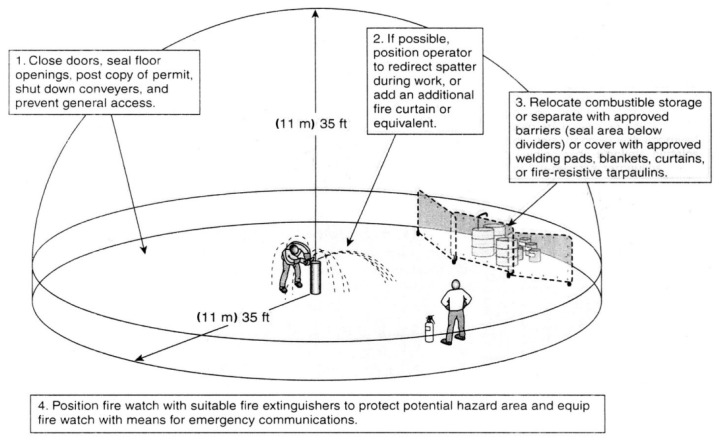
35-ft rule for cutting or welding operation in NFPA 51b [1].

Given the dangerous nature of the aforementioned condition, it is crucial to avoid it as much as possible. The National Fire Protection Association (NFPA) in the US has introduced the NFPA 51b regulation to prevent fire or explosions resulting from hot work projects, including welding, heat treating, grinding, and similar applications producing sparks, flames, or heat. This regulation ensures fire prevention during welding and hot work processes and is recognised in the US and South Korea. NFPA 51b stipulates that there should be no combustible materials within an 11 m (or 35 ft) radius of any hot work, as shown in Figure 2. In South Korea, the Korea Occupational Safety and Health Standards Rules (Article 241) adopts the 11 m rule for welding, cutting, and brazing operations, in accordance with the safety requirements established by NFPA 51b. By adhering to Article 241, most fire incidents on construction sites are likely to be prevented. However, this regulation is often violated by many medium- or small-sized construction sites, leading to repeated catastrophic incidents in South Korea.

This situation gave rise to an idea that the recent advances in computer vision technology might be used to reduce such catastrophic incidents drastically. Object detection is a computer vision technology used to identify target objects in an image. It has the potential to enhance safety on construction sites through remote surveillance, enabling the detection of non-compliance with fire safety regulations.

The field of object detection has witnessed significant development over the past 20 years, typically divided into two distinct periods: the traditional object detection period (prior to 2014) and the deep learning-based detection period (since 2014) [2].

During the traditional object detection period, computer vision engineers relied on handcrafted features such as edges, colours, and simple textures that were distinctive in each given image [3]. The selection of these features was based on the engineers’ judgment and involved a lengthy trial and error process to determine the most effective features for different object classes [3]. Examples are the Viola-Jones detector [4], Histogram of Oriented Gradients (HOG) [5], and Deformable Part-based Model (DPM) [6].

In 2012, AlexNet [7] introduced a multi-GPU training approach, enabling faster training of larger models. Since 2014, object detectors have undergone a rapid evolution by allocating substantial computational resources to the graphics processing unit (GPU) rather than the central processing unit (CPU). In the deep learning-based detection period, object detectors can be categorised as two-stage or one-stage detectors.

Two-stage detectors propose approximate object regions using deep features before performing image classification and bounding box regression. Examples of two-stage detectors include Regions with Convolutional Neural Networks (R-CNN) [8], Spatial Pyramid Pooling network (SPP-Net) [9], Fast R-CNN [10], Faster R-CNN [11], and Feature Pyramid Networks (FPN) [12].

One-stage detectors handle object localisation and classification simultaneously, offering advantages such as fast inference speed, simplicity, and efficiency compared to two-stage detectors. Examples of one-stage detectors include You Only Look Once (Yolo) [13], Single Shot MultiBox Detector (SSD) [14], RetinaNet [15], CenterNet [16], EfficientDet [17], and Deformable Transformers for End-to-End Object Detection (Deformable DETR) [18].

Recent studies have applied object detection to early-stage forest fire detection with high accuracy, distinguishing fire from fire-like objects (e.g., the sun) and detecting even small fires. Additionally, lightweight forest fire detection models have been developed for deployment on hardware devices such as CCTV. These applications typically employ one-stage detectors such as Yolov3, SSD [19,20], Yolov5, EfficientDet [21], Yolov5 [22,23,24], and Deformable DETR [25]. Similarly, object detectors have been employed for fire detection in urban indoor and outdoor environments, including chemical facility fire detection using Yolov2 [26], fire and smoke detection using Yolov3 and Yolov2 [27,28], and indoor fire and smoke detection using Faster R-CNN and Yolov5 [29,30,31].

In the context of safety on construction sites, object detection has been utilised to detect fire ignition sources such as welding sparks and fire safety equipment such as fire extinguishers and fire buckets using models such as Yolov5 [32] and Yolov4 [33]. Although previous research [32,33] has focused on detecting ignition sources like welding sparks on construction sites, it has overlooked a crucial aspect in analyzing combustible materials such as urethane foam and Styrofoam, which possess the potential to escalate fires on a large scale. The research [33] introduced real-time object detection technology for identifying fires on construction sites, but primarily focused on post fire-occurrence detection, without a prevention strategy before an occurrence of fire.

This study aims to detect fire risks by identifying the presence of combustible materials (urethane foam/Styrofoam) and ignition sources (welding sparks) on construction sites. For a rigorous detection of fire risk on construction sites, the distance between an ignition source and a combustible material needs to be identified. However, due to the technical challenge involved in the process, this study focuses only on detecting the coexistence of an ignition source and a combustible material in a single camera view from a construction site using deep learning as the first stage of study.

Two deep learning models, Yolov5 and EfficientDet, were chosen as candidate deep learning models, and their performances were compared for detecting sparks as ignition sources and urethane foam and Styrofoam as combustible materials.

This paper is structured as follows. Section 2 provides an overview of fire incidents on construction sites in South Korea. Section 3 discusses fire detection methods, highlighting their functionalities and characteristics. Section 4 presents a comparison of the performance of these methods. Section 5 shows the experimental results, followed by a conclusion summarising the key findings.

## 2. Fire Incidents on Construction Sites in South Korea

**Figure 3:**
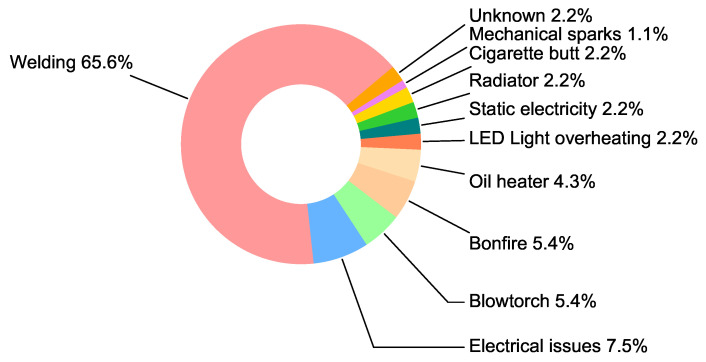
Ignition sources in fire incidents on construction sites in South Korea.

Statistical analysis was carried out to identify the ignition sources and combustible materials commonly found in fire incidents on construction sites in South Korea. A dataset comprising 93 large-scale fire incidents that occurred between 2000 and 2019 was collected from the Korea Occupational Safety and Health Agency (KOSHA). Figure 3 presents an overview of the ignition sources found in the fire incidents, showing the sparks during hot work as the primary cause of fires.

**Figure 4:**
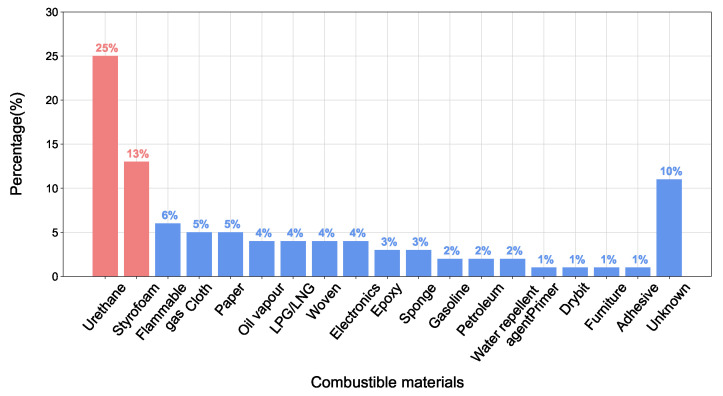
Combustible materials in fire incidents on construction sites.

Figure 4 illustrates the combustible materials typically present on construction sites. Notably, urethane and Styrofoam constituted the majority of combustible materials present in the incidents. It can be seen that the coexistence of ignition sources such as welding sparks and combustible materials such as Styrofoam and urethane foam poses a significant risk of fires on construction sites.

## 3. Object Detection

**Figure 5:**
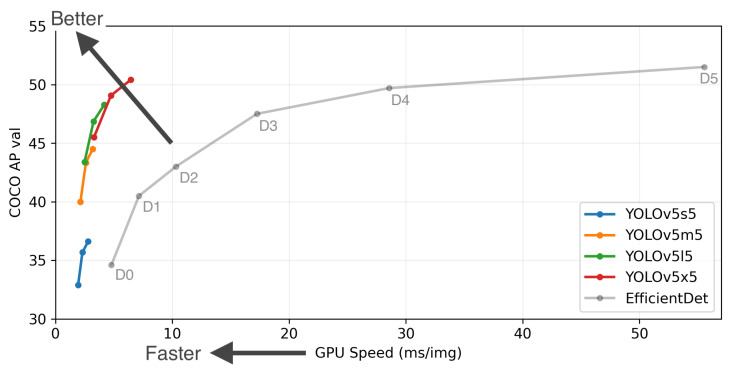
Performance of Yolov5 and EfficietDet [35].

Object detection has gained widespread adoption in various domains, including autonomous driving and video surveillance. Figure 5 shows the performances of the two state-of-the-art object detectors in terms of average precision (AP) on the Microsoft COCO dataset. Yolov5 and EfficientDet have demonstrated exceptional performance on the Microsoft COCO image dataset and have been extensively utilised in real-world applications [34]. Table 1 provides a summary of performance of the two object detectors on custom datasets. Yolov5 tends to have a slightly better performance than EfficientDet.

### 3.1. Yolov5

Yolov5 is a powerful state-of-the-art one-stage object detector [21]. Its architecture comprises three parts: (1) Backbone: CSPDarknet, (2) Neck: PANet, and (3) Head: Yolo Layer [21]. Compared to Yolov4, Yolov5 is significantly smaller, with a size of approximately 27 MB instead of 244 MB. It also offers faster inference times, achieving around 140 frames per second (FPS) compared to Yolov4’s 50 FPS on the Tesla P100 GPU, while maintaining comparable mean Average Precision (mAP) performance.

Yolov5 offers five types of neural networks depending on the complexity of the network (see Table 2). Yolov5n is the smallest and fastest neural network, suitable for various applications. Yolov5n/s/m are designed for mobile deployments, while Yolov5l/x are intended for cloud deployments. Larger models like Yolov5l and Yolov5x generally deliver better results across different scenarios but have more parameters, require more CUDA memory for training, and exhibit slower inference speeds.

### 3.2. EfficientDet

EfficientDet is an advanced object detector developed by the Google Brain Team, consistently outperforming previous approaches in terms of efficiency under various resource constraints. The architecture of EfficientDet comprises three main components: (1) Backbone: EfficientNet, (2) Neck: BiFPN, and (3) Head. One of the key features of EfficientDet is the utilization of feature fusion techniques through a bidirectional feature pyramid network (BiFPN), which combines representations of input images at different resolutions [37]. This approach enables EfficientDet to achieve high accuracy with fewer parameters and high floating-point operations per second (FLOPS) [21]. EfficientDet offers pre-trained weights categorised from D0 to D7, with D0 having the fewest parameters and D7 having the highest number of parameters [37].

## 4. Fire Risk Detection by Object Detection

### 4.1. Dataset Preparation

The image dataset used in this study comprised images and videos of welding sparks, urethane foam, and Styrofoam sourced from Google and Naver search engines, as well as images obtained from the Korean AI integration platform (https://aihub.or.kr, accessed on 21 January 2023). Low-resolution or irrelevant images were removed manually from the search results. The numbers of images used in four trials in this study is shown in Table 3. In order to achieve the maximum performance, four different model training trials were carried out as discussed below.

### 4.2. Image Labeling Approach

Each image in the image dataset had to be labeled with bounding boxes to be used as training, validation, or test datasets. Typically, object detection was used to detect objects with a distinct shape, such as people, cups, or trees. However, object detection on sparks and urethane foam generally poses a challenge, as their shapes are not well-defined. For example, the shape of a spark depends on how it is generated, i.e., welded, flame-cut, or ground, and the shape of urethane foam depends on the specific spot where it is sprayed. This creates an uncertainty around how to label images for sparks and urethane foam. In addition, Styrofoam is prone to partial occlusion when stacked on construction sites.

Different image labeling approaches were explored and their Average Precision (AP) values were compared to determine the best approach. AP values were calculated using Yolov5s.

#### 4.2.1. Sparks

**Figure 6:**
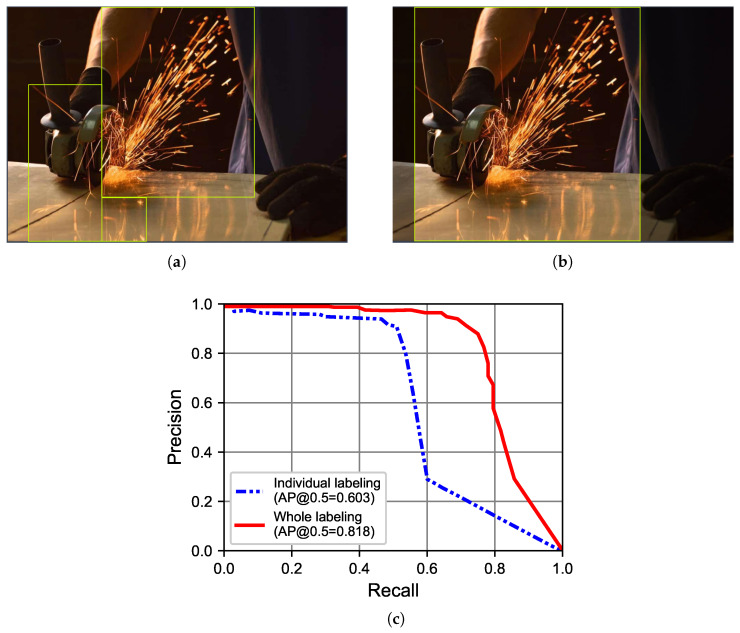
Two labeling approaches and their performance on sparks. (**a**) Individual labeling. (**b**) Whole labeling. (**c**) AP.

For labeling images of sparks, two different labeling approaches were used: individual labeling and whole labeling, as shown in Figure 6. The individual labeling approach assigns multiple bounding boxes to each image, as shown in Figure 6a, where the image was labeled with three bounding boxes. The whole labeling approach assigns a single bounding box to cover all the sparks, as shown in Figure 6b.

With 1900 images, training adopted a 6:2:2 ratio for training, validation, and test datasets in Table 3. This yielded an average precision (AP) of 60.3% for individual and 81.8% for whole labeling (Figure 6c). Notably, whole labeling outperformed individual labeling for sparks.

#### 4.2.2. Urethane Foam

**Figure 7:**
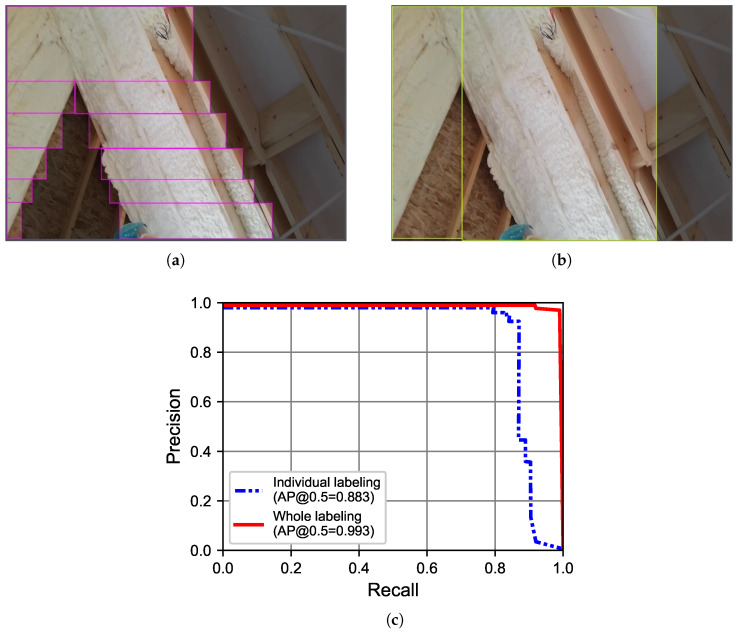
Two labeling approaches and their performance on urethane foam. (**a**) Individual labeling. (**b**) Whole labeling. (**c**) AP.

The same individual and whole labeling approaches were used for urethane foam. The individual labeling approach involved using more than 10 small bounding boxes per image, as shown in Figure 7a. The whole labeling approach employed 2–3 large bounding boxes per image, as shown in Figure 7b. Using 114 images, a 6:2:2 split ratio was used for training, validation, and test datasets (Table 3). Figure 7c shows average precision (AP) for urethane foam, comparing individual and whole labeling results.

The AP achieved through individual labeling for urethane foam was 88.3%, while the AP for the whole labeling approach was 93.3%. The improvement in AP for the whole labeling approach can be attributed to the larger bounding box size. Therefore, to achieve a higher AP, it is important to include as much of the urethane foam area as possible within a bounding box.

#### 4.2.3. Styrofoam

**Figure 8:**
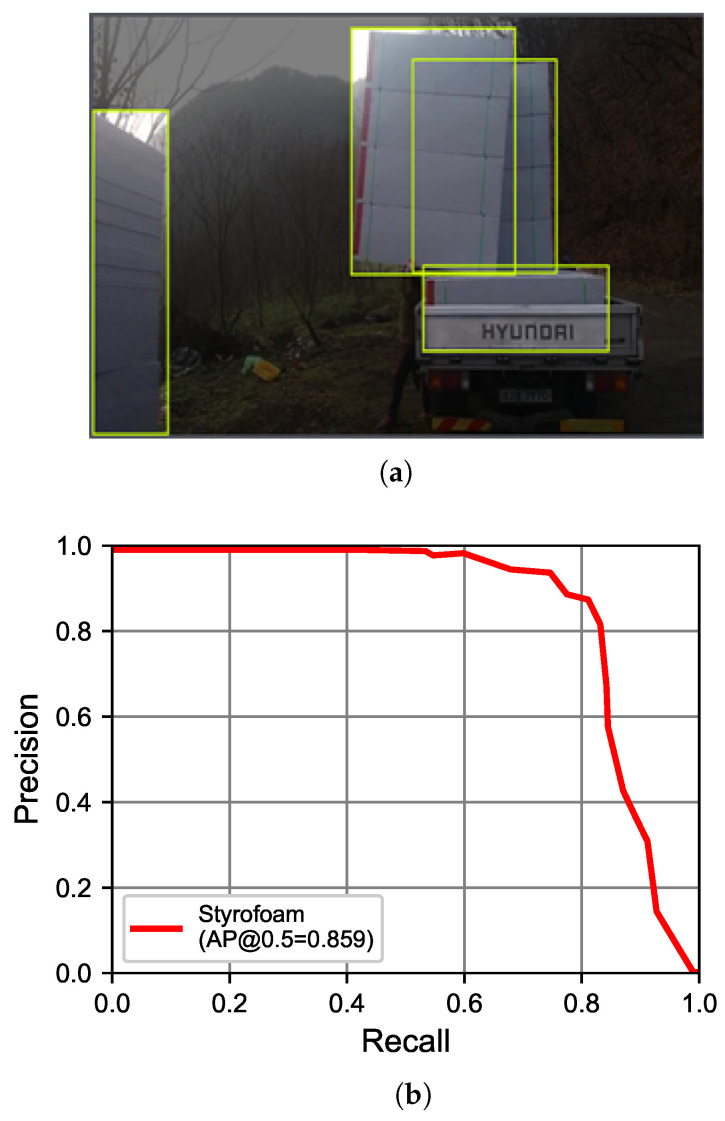
Labeling approach and its performance on Styrofoam. (**a**) Labeling. (**b**) AP.

Styrofoam is frequently stacked in bulk quantities on construction sites, often leading to partial occlusion of the material. When labeling Styrofoam, it is generally considered the best practice to label the occluded object as if it were fully visible, rather than drawing a bounding box solely around the partially visible portion as shown in Figure 8a. Training with 1381 Styrofoam images used a 6:2:2 ratio for training, validation, and test datasets (Table 3), achieving an AP of 85.9% in Figure 8b.

### 4.3. Long-Distance Object Detection

The image dataset used so far only consists of images of near objects. However, in real applications, it is ideal to be able to detect objects at further distances. The performance of the object detector for a long-distance object will be discussed in this section.

For the Yolov5s model trained, its performance for long-distance objects was calculated using a new test dataset containing only long-distance images. To enhance the detection performance for long-distanced objects, additional long-distance images were added to the training, validation, and test datasets. The model was then retrained using the updated image dataset, and its performance was evaluated on the updated test dataset.

#### 4.3.1. Sparks

**Figure 9:**
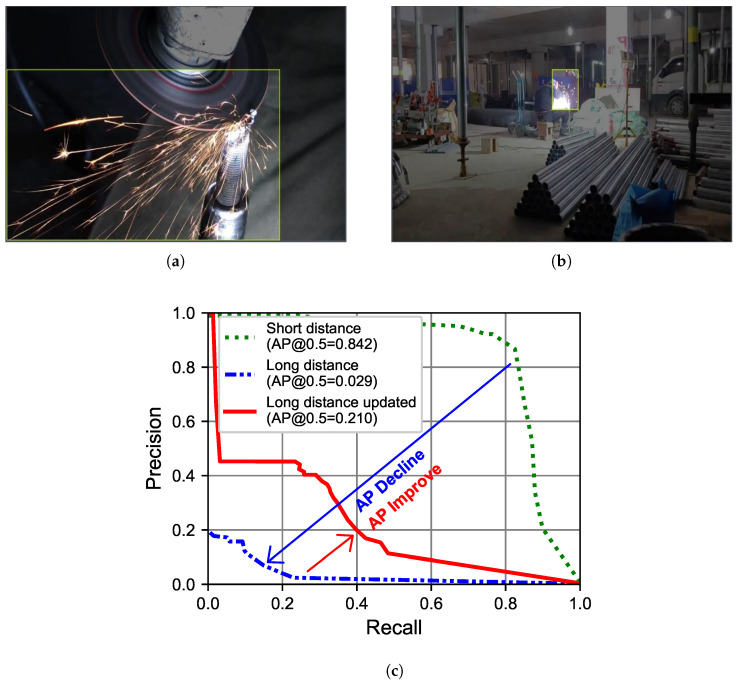
Short- and long-distance labeling approaches used for sparks. (**a**) Short-distance labeling. (**b**) Long-distance labeling. (**c**) Performance.

The training dataset comprised 1520 images, with a split of 6:2:2 (training:validation:test) as shown in Table 3, focusing on short-distance sparks (Figure 9a). A model trained solely on these images achieved an AP of 84.2% on the short-distance test dataset.

For performance evaluation on long-distance images, the test dataset was replaced by new 304 long-distance images (Figure 9b) and the AP value was evaluated, resulting in an AP of 2.9% significantly lower the original AP of 84.2%, as shown in Figure 9c.

To enhance long-distance spark detection, 330 long-distance images were added (6:2 ratio) to training: validation datasets (Table 3). This improved test dataset performance from 2.9% to 21% as shown in Figure 9c.

#### 4.3.2. Urethane Foam

**Figure 10:**
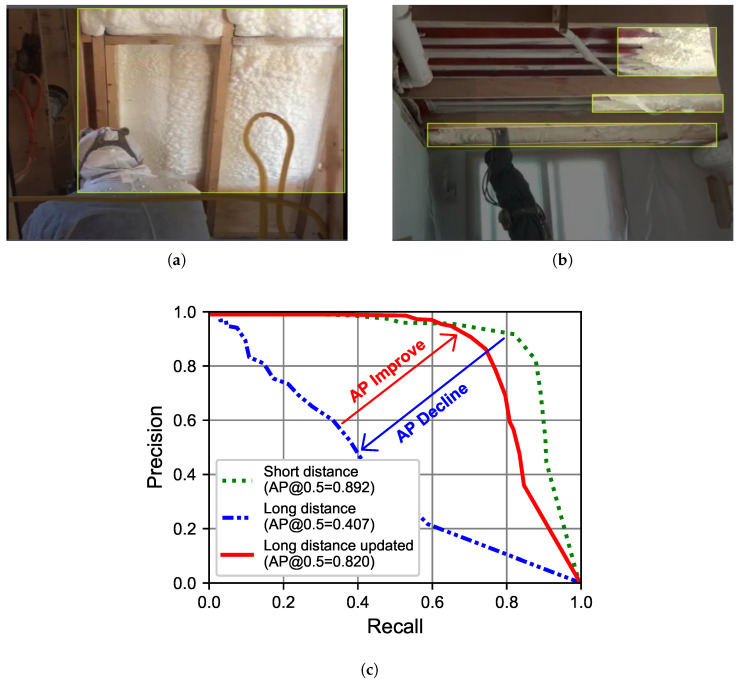
Short- and long-distance labeling approaches used for urethane foam. (**a**) Short-distance labeling. (**b**) Long-distance labeling. (**c**) Performance.

The dataset contains 1518 short-distance images (Figure 10a). These were split with a ratio of 6:2:2 (training:validation:test datasets), as shown in Table 3. After training on short-distance urethane foam images, an AP of 89.2% was achieved. When substituting short-distance test images with long-distance 304 urethane foam images (Figure 10b) in the test dataset, the model achieved a lower AP of 40.7%, as shown in Figure 10c.

#### 4.3.3. Styrofoam

**Figure 11:**
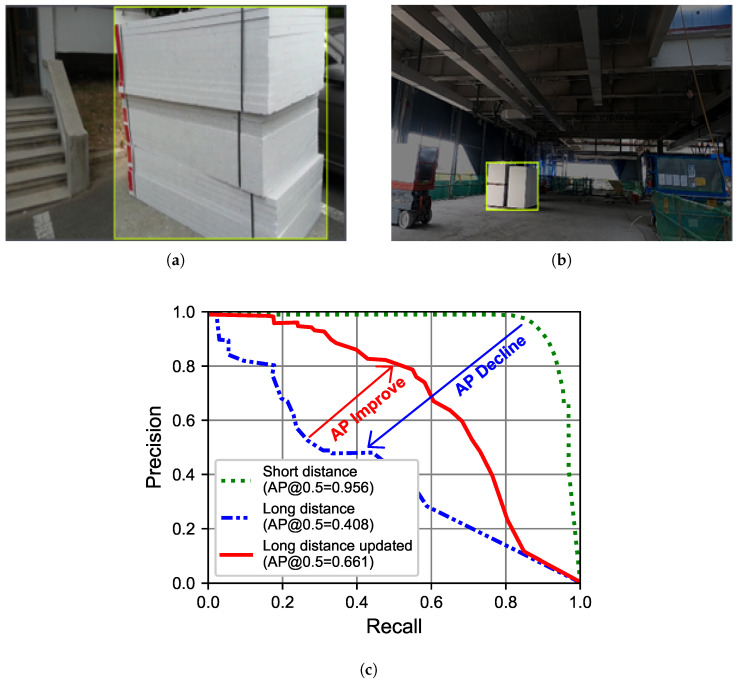
Short- and long-distance labeling approaches used for Styrofoam. (**a**) Short-distance labeling. (**b**) Long-distance labeling. (**c**) Performance.

The dataset of 824 images was divided with a ratio of 6:2:2 into training:validation:test datasets, as shown in Table 3. The model, trained on short-distance Styrofoam images (Figure 11a), attained a 95.6% AP on the 163 images of short-distance test dataset (Figure 11c). However, its performance dropped to 40.8% AP when tested on 163 long-distance Styrofoam images (Figure 11b).

Again, to enhance long-distance detection, 385 long-distance Styrofoam images were added (6:2 ratio) to (training:validation) datasets (Table 3). This increased AP from 40.8% to 66.1% (Figure 11c).

To ensure better performance of long-distance object detection, it is of paramount importance that enough long-distance images are included in the dataset.

### 4.4. Performance of Yolov5 and EfficientDet

**Figure 12:**
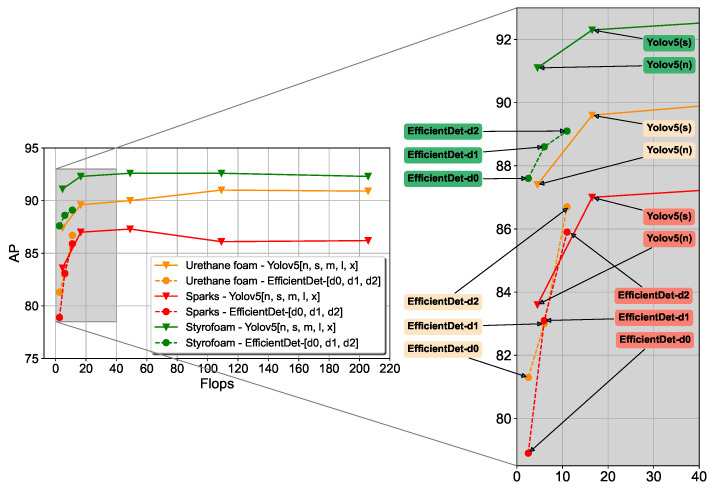
Performance comparison of Yolov5 and EfficientDet networks on sparks, urethane foam, and Styrofoam.

**Figure 13:**
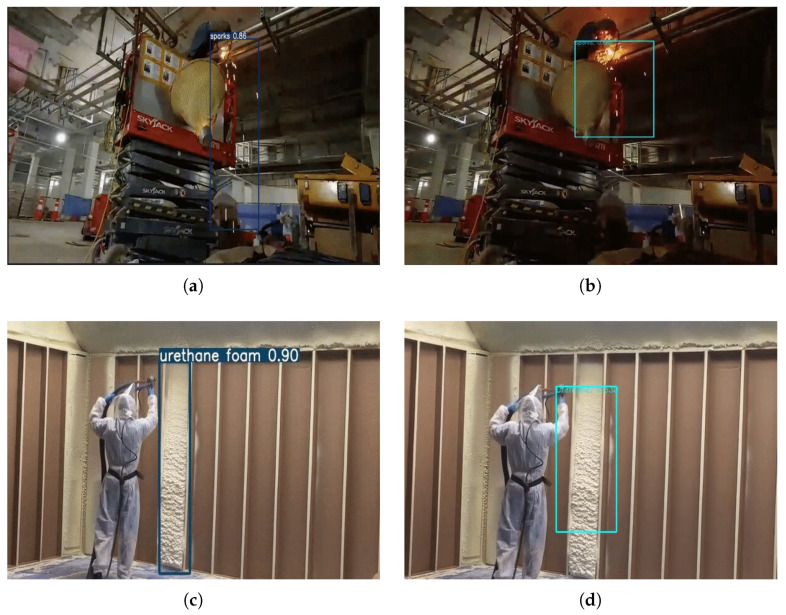

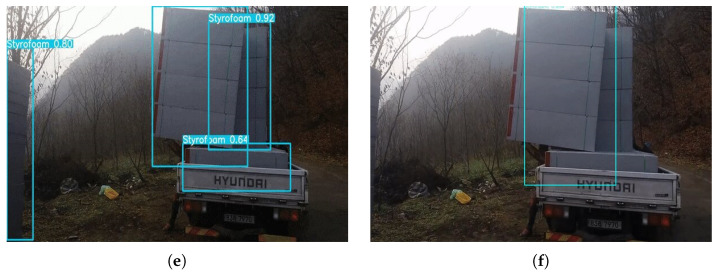
Typical detection result of the trained Yolov5 or EfficientDet. (**a**) Sparks detection (Yolov5s). (**b**) Sparks detection (EfficientDet-d1). (**c**) Urethane foam detection (Yolov5s). (**d**) Urethane foam detection (EfficientDet-d1). (**e**) Styrofoam detection (Yolov5s). (**f**) Styrofoam detection (EfficientDet-d1).

The performance of Yolov5 and EfficientDet was compared in the final dataset, as shown in Table 3. The dataset was constructed using the whole labeling approach and also includes short-, medium-, and long-distanced images. Different sized models of Yolov5 and EfficientDet were all trained and their performance was evaluated, as shown in Figure 12 and Figure 13 and Table 4. Yolov5 models were found to have slightly better APs from 87% to 90% than EfficientDet models from 82 % to 87%. However, it was found that Yolov5 was easier to train than EfficientDet, reaching convergence without the need for tuning parameters such as learning rate, batch size, and a choice of optimization algorithm.

In addition, it should be noted that EfficientDet tends to scale up image size, resulting in higher memory consumption and slower training [46]. On the other hand, Yolov5’s architecture is lightweight, allowing training with smaller computational resources and cost-effectiveness.

**Figure 14:**
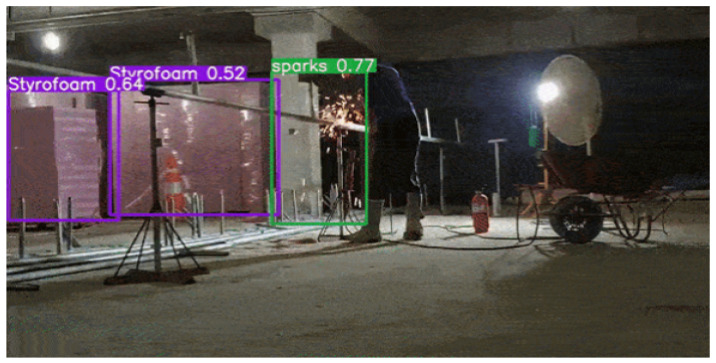
Example of fire risk detection on the construction site.

Figure 14 shows an example of fire risk detection on construction sites where Styrofoam is in close proximity to welding sparks. The trained Yolov5s model was found to successfully identify sparks and Styrofoams at the same time in a single camera view. The developed fire risk detection model may be used as a proactive fire risk management tool on construction sites.

## 5. Conclusions

To reduce catastrophic fire incidents on construction sites in South Korea, object detection technology was employed for detecting the fire risk that an ignition source and a combustible material coexist in a single-camera view of a surveillance camera on a construction site. Two candidate deep learning models, Yolov5 and EfficientDet, were compared on their performance in detecting welding sparks (as an ignition source) and urethane foam and Styrofoam (as combustible materials).

- Improved Labeling for Enhanced Performance: To maximise the performance of the deep learning models in terms of the mean average precision (mAP), for detecting fire risks such as sparks and urethane foam, it was observed that higher mAPs were achieved by the labeling approach that encompassed the entire object(s) with relatively large bounding box(es). This improved labeling approach significantly improved the detection performance mAPs by around 15% for the given dataset.
- Improved Long-Distance Object Detection: To enhance long-distance object detection, the study highlighted the importance of inclusion of images from diverse scenarios with varying distances into the dataset. By incorporating long-distance images, the model’s ability to detect fire risks was notably improved, increasing the detection performance mAP by around 28% for the given dataset.
- Best Model for Fire Risk Detection: In terms of the fire risk detection performance, Yolov5 showed a slightly better performance than EfficientDet for the given set of objects—sparks, urethane foam, and Styrofoam. It was found that YOLOv5 was easier to train without the need to fine-tune hyperparameters such as learning rate, batch size, and a choice of optimization algorithm.

Future work will concentrate on enhancing fire risk detection by incorporating the distance between combustible materials and ignition sources. Utilising depth estimation to measure these distances will yield valuable insights into the level of fire risks. By classifying the level of fire risk based on distance, a more quantitative assessment of fire risks can be achieved on construction sites. After the successful detection of fire risk using the proposed approach, an alarm can be notified to safety managers on the construction site or fire safety authorities, which can initiate appropriate action to manage the risk identified.

## Figures and Tables

**Table 1:** State-of-the-art object detection performance based on Yolov5, EfficientDet.

Title of Research Article	Year	Object	# of Images	Object Detector (AP)	
A Forest Fire Detection System Based on Ensemble Learning [21]	2021	Forest Fire	2381	EfficientDet (0.7570)	Yolov5 (0.7050)
Garbage Detection using Advanced Object Detection Techniques [36]	2021	Garbage	500	EfficientDet-D1 (0.3600)	Yolov5m (0.6130)
Deep Learning in Diabetic Foot Ulcers Detection: A Comprehensive Evaluation [37]	2020	Ulcers	2000	EfficientDet (0.5694)	Yolov5 (0.6294)
A Real-Time Apple Targets Detection Method for Picking Robot Based on Improved Yolov5 [38]	2021	Apple	1214	EfficientDet-D0 (0.8000)	Yolov5s (0.8170)
Research on Detecting Bearing-Cover Defects Based on Improved Yolov3 [39]	2021	Bearing-Cover	1995	EfficientDet-D2 (0.5630)	Yolov5 (0.5670)
A first step towards automated species recognition from camera trap images of mammals using AI in a European temperate forest [40]	2021	Mammals	2659	Yolov5 (0.8800)	
An Application of Deep-Learning Techniques to Face Mask Detection During the COVID-19 Pandemic [41]	2021	Face masks	848	Yolov5 (0.8100)	
Toward More Robust and Real-Time Unmanned Aerial Vehicle Detection and Tracking via Cross-Scale Feature Aggregation Based on the Center Key point [42]	2021	Drones	5700	Yolov5 (0.9690)	
Towards automatic waste containers management in cities via Computer Vision: containers localization and geo-positioning in city maps [43]	2022	Waste containers	2381	EfficientDet (0.8400)	Yolov5 (0.8900)
Performance evaluation of deep learning object detectors for weed detection for cotton [44]	2022	Weed	5187	EfficientDet-D2 (0.7783)	Yolov5n (0.7864)
Detecting broiler chickens on litter floor with the YOLOv5-CBAM deep learning model [45]	2023	Chickens	560	EfficientDet (0.5960)	Yolov5s (0.9630)

**Table 2:** Details of Yolov5 neural networks [35].

Model	Size (Pixels)	Params (M)	FLOPs (B)	mAP@0.5 ^1^ (%)
Yolov5n	640	1.9	4.5	45.7
Yolov5s	640	7.2	16.5	56.8
Yolov5m	640	21.2	49.0	64.1
Yolov5l	640	46.5	109.1	67.3
Yolov5x	640	86.7	205.7	68.9

^1^ mAP scores on COCO dataset.

**Table 3:** Image datasets used in the study.

Object Detection Dataset	Dataset Split Ratios	The Number of Images		
	Training/Validation/Test	Sparks	Urethane Foam	Styrofoam
Image Labeling Dataset	60%/20%/20%	1900	114	1381
Short Distanced Dataset	60%/20%/20%	1520	1518	824
Long Distanced Updated Dataset	63%/21%/16%	1850	2054	1209
Final Dataset	60%/20%/20%	2158	3316	3915

**Table 4:** Performance of Yolov5 and EfficientDet models.

Model	Model’s Performance			
	Sparks AP (%)	Urethane Foam AP (%)	Styrofoam AP (%)	mAP (%)
Yolov5n	83.6	87.4	91.1	87.4
Yolov5s	87.0	89.6	92.3	89.6
Yolov5m	87.3	90.0	92.6	90.0
Yolov5l	86.1	91.0	92.6	89.9
Yolov5x	86.2	90.9	92.3	89.8
EfficientDet-d0	78.0	81.3	87.6	82.3
EfficientDet-d1	83.1	83.0	88.6	84.9
EfficientDet-d2	85.9	86.7	89.1	87.2

## Data Availability

The data presented in this study may be available on request from the corresponding author. The data are not publicly available due to the agreement of the research project.

## References

[1] National Fire Protection Association (2009). NFPA 51B: Standard for Fire Prevention During Welding, Cutting, and Other Hot Work.

[2] Zou Z., Chen K., Shi Z., Guo Y., Ye J. (2023). Object Detection in 20 Years: A Survey. arXiv.

[3] O’Mahony N., Campbell S., Carvalho A., Harapanahalli S., Hernandez G.V., Krpalkova L., Riordan D., Walsh J. Deep Learning vs. Traditional Computer Vision. Proceedings of the 2019 Computer Vision Conference (CVC).

[4] Viola P., Jones M.J. (2004). Robust Real-Time Face Detection. Int. J. Comput. Vis..

[5] Dalal N., Triggs B. Histograms of oriented gradients for human detection. Proceedings of the 2005 IEEE Computer Society Conference on Computer Vision and Pattern Recognition (CVPR’05).

[6] Felzenszwalb P., McAllester D., Ramanan D. A discriminatively trained, multiscale, deformable part model. Proceedings of the 2008 IEEE Conference on Computer Vision and Pattern Recognition.

[7] Krizhevsky A., Sutskever I., Hinton G.E. (2017). ImageNet classification with deep convolutional neural networks. Commun. ACM.

[8] Girshick R., Donahue J., Darrell T., Malik J. (2014). Rich feature hierarchies for accurate object detection and semantic segmentation. arXiv.

[9] He K., Zhang X., Ren S., Sun J. Spatial Pyramid Pooling in Deep Convolutional Networks for Visual Recognition. Proceedings of the 13th European Conference.

[10] Girshick R. (2015). Fast R-CNN. arXiv.

[11] Ren S., He K., Girshick R., Sun J. (2016). Faster R-CNN: Towards Real-Time Object Detection with Region Proposal Networks. arXiv.

[12] Lin T.Y., Dollár P., Girshick R., He K., Hariharan B., Belongie S. (2017). Feature Pyramid Networks for Object Detection. arXiv.

[13] Redmon J., Divvala S., Girshick R., Farhadi A. (2016). You Only Look Once: Unified, Real-Time Object Detection. arXiv.

[14] Liu W., Anguelov D., Erhan D., Szegedy C., Reed S., Fu C.Y., Berg A.C. SSD: Single Shot MultiBox Detector. Proceedings of the Computer Vision-ECCV 2016.

[15] Lin T.Y., Goyal P., Girshick R., He K., Dollár P. (2018). Focal Loss for Dense Object Detection. arXiv.

[16] Zhou X., Wang D., Krähenbühl P. (2019). Objects as Points. arXiv.

[17] Tan M., Pang R., Le Q.V. (2020). EfficientDet: Scalable and Efficient Object Detection. arXiv.

[18] Zhu X., Su W., Lu L., Li B., Wang X., Dai J. (2021). Deformable DETR: Deformable Transformers for End-to-End Object Detection. arXiv.

[19] Wu S., Guo C., Yang J. (2020). Using PCAand one-stage detectors for real-time forest fire detection. J. Eng..

[20] Nguyen A.Q., Nguyen H.T., Tran V.C., Pham H.X., Pestana J. A Visual Real-time Fire Detection using Single Shot MultiBox Detector for UAV-based Fire Surveillance. Proceedings of the 2020 IEEE Eighth International Conference on Communications and Electronics (ICCE).

[21] Xu R., Lin H., Lu K., Cao L., Liu Y. (2021). A Forest Fire Detection System Based on Ensemble Learning. Forests.

[22] Wei C., Xu J., Li Q., Jiang S. (2022). An Intelligent Wildfire Detection Approach through Cameras Based on Deep Learning. Sustainability.

[23] Xue Z., Lin H., Wang F. (2022). A Small Target Forest Fire Detection Model Based on YOLOv5 Improvement. Forests.

[24] Mukhiddinov M., Abdusalomov A.B., Cho J. (2022). A Wildfire Smoke Detection System Using Unmanned Aerial Vehicle Images Based on the Optimized YOLOv5. Sensors.

[25] Huang J., Zhou J., Yang H., Liu Y., Liu H. (2023). A Small-Target Forest Fire Smoke Detection Model Based on Deformable Transformer for End-to-End Object Detection. Forests.

[26] Wu H., Wu D., Zhao J. (2019). An intelligent fire detection approach through cameras based on computer vision methods. Process. Saf. Environ. Prot..

[27] Li P., Zhao W. (2020). Image fire detection algorithms based on convolutional neural networks. Case Stud. Therm. Eng..

[28] Saponara S., Elhanashi A., Gagliardi A. (2021). Real-time video fire/smoke detection based on CNN in antifire surveillance systems. J.-Real-Time Image Process..

[29] Pincott J., Tien P.W., Wei S., Kaiser Calautit J. (2022). Development and evaluation of a vision-based transfer learning approach for indoor fire and smoke detection. Build. Serv. Eng. Res. Technol..

[30] Pincott J., Tien P.W., Wei S., Calautit J.K. (2022). Indoor fire detection utilizing computer vision-based strategies. J. Build. Eng..

[31] Ahn Y., Choi H., Kim B.S. (2023). Development of early fire detection model for buildings using computer vision-based CCTV. J. Build. Eng..

[32] Dwivedi U.K., Wiwatcharakoses C., Sekimoto Y. Realtime Safety Analysis System using Deep Learning for Fire Related Activities in Construction Sites. Proceedings of the 2022 International Conference on Electrical, Computer, Communications and Mechatronics Engineering (ICECCME).

[33] Kumar S., Gupta H., Yadav D., Ansari I.A., Verma O.P. (2022). YOLOv4 algorithm for the real-time detection of fire and personal protective equipments at construction sites. Multimed. Tools Appl..

[34] Everingham M., Eslami S.M.A., Van Gool L., Williams C.K.I., Winn J., Zisserman A. (2015). The Pascal Visual Object Classes Challenge: A Retrospective. Int. J. Comput. Vis..

[35] Jocher G., Chaurasia A., Stoken A., Borovec J., NanoCode012, Kwon Y., Michael K., TaoXie, Fang J., imyhxy (2022). ultralytics/yolov5: V7.0—YOLOv5 SOTA Realtime Instance Segmentation. Programmers: _:n2611. https://zenodo.org/records/7347926.

[36] Patel D., Patel F., Patel S., Patel N., Shah D., Patel V. Garbage Detection using Advanced Object Detection Techniques. Proceedings of the 2021 International Conference on Artificial Intelligence and Smart Systems (ICAIS).

[37] Yap M.H., Hachiuma R., Alavi A., Brungel R., Cassidy B., Goyal M., Zhu H., Ruckert J., Olshansky M., Huang X. (2021). Deep Learning in Diabetic Foot Ulcers Detection: A Comprehensive Evaluation. arXiv.

[38] Yan B., Fan P., Lei X., Liu Z., Yang F. (2021). A Real-Time Apple Targets Detection Method for Picking Robot Based on Improved YOLOv5. Remote. Sens..

[39] Zheng Z., Zhao J., Li Y. (2021). Research on Detecting Bearing-Cover Defects Based on Improved YOLOv3. IEEE Access.

[40] Choinski M., Rogowski M., Tynecki P., Kuijper D.P.J., Churski M., Bubnicki J.W. (2021). A first step towards automated species recognition from camera trap images of mammals using AI in a European temperate forest. arXiv.

[41] Khamlae P., Sookhanaphibarn K., Choensawat W. An Application of Deep-Learning Techniques to Face Mask Detection During the COVID-19 Pandemic. Proceedings of the 2021 IEEE 3rd Global Conference on Life Sciences and Technologies (LifeTech).

[42] Bao M., Chala Urgessa G., Xing M., Han L., Chen R. (2021). Toward More Robust and Real-Time Unmanned Aerial Vehicle Detection and Tracking via Cross-Scale Feature Aggregation Based on the Center Keypoint. Remote. Sens..

[43] Moral P., García-Martín Á., Escudero-Viñolo M., Martínez J.M., Bescós J., Peñuela J., Martínez J.C., Alvis G. (2022). Towards automatic waste containers management in cities via computer vision: Containers localization and geo-positioning in city maps. Waste Manag..

[44] Rahman A., Lu Y., Wang H. (2023). Performance evaluation of deep learning object detectors for weed detection for cotton. Smart Agric. Technol..

[45] Guo Y., Aggrey S.E., Yang X., Oladeinde A., Qiao Y., Chai L. (2023). Detecting broiler chickens on litter floor with the YOLOv5-CBAM deep learning model. Artif. Intell. Agric..

[46] Tan M., Le Q.V. (2021). EfficientNetV2: Smaller Models and Faster Training. arXiv.

